# Multinucleation in Day Two Embryos Is Not Associated with Multinucleation in Sibling Embryos After Freezing and Thawing

**Published:** 2020

**Authors:** Jaana Seikkula, Päivi Polo-Kantola, Harri Mankonen, Leena Anttila, Varpu Jokimaa

**Affiliations:** 1- Department of Obstetrics and Gynecology, Central Finland Central Hospital, Jyväskylä and University of Turku, Turku, Finland; 2- Department of Obstetrics and Gynecology, University of Turku and Turku University Hospital, Turku, Finland

**Keywords:** Cell nucleus, Cryopreservation, ICSI, IVF

## Abstract

**Background:**

Multinucleated embryos exhibit impaired implantation potential, but whether the presence of multinucleated embryos in an embryo cohort reflects the quality of the entire cohort is controversial. No data exists on multinucleation rate among frozen-thawed embryos.

**Methods:**

De novo multinucleation and the number of multinucleated embryos on day two of embryo culture before freezing (D2) (n=415), at thawing (D2t) (n=320) and after an overnight culture after thawing (D3t) (n=265) was recorded. Associations between multinucleation before and after cryopreservation, female age and ovarian sensitivity to hormonal stimulation were assessed.

**Results:**

The occurrence of at least one multinucleated embryo per embryo cohort was 62.4% on D2, 16.3% on D2t and 31.7% on D3t. The presence of multinucleated embryos prior to freezing was not associated with de novo multinucleation during post-thaw culture (p=0.845). On D2, multinucleation was high in young women, irrespective of the number of collected oocytes (p=0.702). In older age groups, multinucleation was highest if >17 oocytes were obtained (p<0.001) and the odds for multinucleation was the lowest if the consumption of recombinant follicle-stimulating hormone was >238 *IU/oocyte* (In the age group of 30–35 years OR 0.25 [0.13–0.47], and the age group of 36–40 years OR 0.35 [0.20–0.63].

**Conclusion:**

Multinucleation is commonly seen in embryos and good-quality day two embryo cohorts before freezing. The presence of multinucleated embryos prior to freezing does not illustrate multinucleation in sibling embryos after thawing. Embryo multinucleation is associated with factors related to good prognosis in assisted reproduction treatments.

## Introduction

Blastomere multinucleation is considered a sign of impaired embryo quality. It is suggested to be originated from abnormalities during oocyte maturation and early embryo development via several mechanisms that result in cytokinetic and/or chromosomal changes in the embryo (1–3). Literature on clinical factors predisposing embryos to development of blastomere multinucleation is inconsistent. Ovarian sensitivety to gonadotropin treatment is reported to be associated with multinucleated (MN) embryos in *in vitro* fertilization (IVF) and intracytoplasmic sperm injection (ICSI) cycles (4–7), but literature on the association between female age and MN embryos is conflicting (6–11). Cooling of oocytes is shown to induce spindle disorganization; therefore, exposure to low temperatures, such as during cryopreservation, might induce formation of multinucleation via negative impact on spindle function and integrity (12, 13). However, data on occurrence of MN embryos after thawing is limited (14).

MN embryos have been reported to possess reduced implantation capacity due to increased aneuploidy rate and they are commonly discarded from transfer and cryopreservation (4, 6, 15, 16). In addition, the pregnancy potential of the sibling embryos has also been debated (5, 7, 8, 17). It is of interest whether the presence of MN embryos before freezing illustrates the quality of the entire embryo cohort in terms of eligibility for freezing and de novo formation of MN embryos after thawing. Furthermore, the identification of various clinical factors that may increase the occurrence of MN embryos after thawing, such as female age and gonadotropin dose, warrants evaluation.

In this study, the rate of de novo formation of MN embryos after thawing of the cryopreserved embryos was evaluated. Further, association between pre-freeze MN embryo formation and occurrence of blastomere multinucleation in postthaw MN embryos was studied. In addition, an attempt was made to assess whether female age and ovarian sensitivity to hormonal stimulation were associated with MN embryo formation before freezing or after thawing.

## Methods

**Figure 1.**
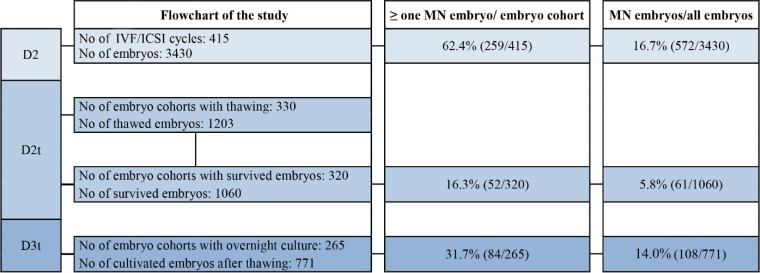
Number of IVF/ICSI cycles and de novo occurence of multinucleation in embryo cohorts and embryos at each time point of evaluation. Colored boxes indicate the analyzed data at the specific time points of evaluation; D2: day of embryo culture; D2t: day of thawing; D3t: day after thawing and an overnight culture

In this study, 415 consecutive IVF/ICSI cycles were analyzed. The cycles included embryo cryopreservation by slow freezing from January 2010 to December 2012 and the frozen embryo transfers until the end of April 2014 at the University Hospital of Turku, Turku, Finland (Figure 1).

### Controlled ovarian stimulation (COS):

Among the 415 IVF/ICSI cycles, pituitary down-regulation was achieved in 227 cycles with gonadotropin releasing hormone agonist protocol and in 183 cycles with antagonist protocol (Data missing in five cycles). Recombinant follicle-stimulating hormone (FSH), human menopausal gonadotropin, or corifollitropin alfa were used for ovarian stimulation.

### Fertilization, embryo culture and assessment:

In 227 cycles, the oocytes were inseminated; in 96 cycles, ICSI was performed and in 92 cycles, both fertilization methods were utilized. Embryos were cultured in a sequential medium of 7% CO_2_, 8% O_2,_ and 85% N_2_ in single embryo droplets at 37± 0.1*°C* and assessed approximately 44 *hr* after fertilization (Day two, D2). Embryos were visualized simultaneously by two experienced observers, one via an inverted microscope (Nikon Diaphot 300) with 400X magnification and the other via a connected PC monitor and, if needed, embryo screening was repeated to reach a consensus.

**Figure 2.**
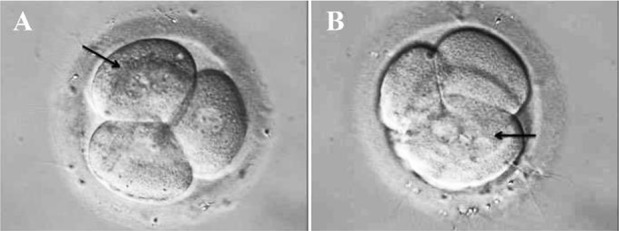
A) Two nuclei in a blastomere (black arrow); B) Multiple nuclei in a blastomere (black arrow)

The embryos were evaluated for blastomere number and evenness, fragmentation and mono/multinuclearity at three time-points: before freezing (D2), within one hour after thawing (D2t) and 23–25 *hr* after thawing (D3t). An embryo was considered multinucleated if two or more nuclei were detected in at least one blastomere (Figure 2). All MN embryos on D2 and D2t were excluded from further analysis and thus, the MN embryos observed after thawing and overnight culture (D3t) reflected de novo findings.

### Embryo freezing:

Embryos with less than 25% fragmentation or difference in blastomere size were considered suitable for freezing. The embryos were frozen 44–46 *hr* after fertilization by using a slow freezing Sydney IVF Cryopreservation Kit (K-SICS-5000, Cook Medical, Australia) according to the manufacturers’ protocol. Cooling was controlled by a freezer (Planer Cryo 10-MRV, UK), starting at 20*°C* and decreasing 3*°C* per minute. Before and after seeding with pre-cooled forceps at −7.0*°C*, a 10 *min* holding period was performed. The temperature was lowered to −30*°C* at 0.3*°C* per minute and to −150*°C* at 50*°C* per minute. The straws were then transferred to liquid N_2_.

### Thawing procedure:

The straws were kept at room temperature (RT) for 40 *s* and thereafter incubated for 30 *s* in a 30*°C* water bath. The embryos were incubated in a series of decreasing cryoprotectant concentrations according to the manufacturer’s protocol (K-SICM-50, Cook Medical, Australia). Subsequently, the embryos were incubated for 5 *min* in a cryopreservation buffer at RT and thereafter the dish was placed for 5 *min* on a heated stage at 39*°C*. The embryos were cultured in a Sydney IVF cleavage medium overnight in 7% CO_2_, 8% O_2_ and 85% N_2_ at 37±0.1*°C*.

### Clinical parameters:

The age of the women, method of fertilization, stimulation protocol used, and the number of oocytes retrieved were recorded and the consumption of recombinant FSH or human menopausal gonadotropin per cycle, as well as the proportional dose of FSH per obtained oocyte, was calculated. Cycles stimulated with corifollitropin alfa (n=25) were excluded from the analysis of FSH consumption, since dosages are not comparable with other preparations. To analyze the associations between MN embryos, female age and ovarian response, the cycles were divided into three groups according to the age of the women (20–29 years, 30–35 years and 36–40 years). The cycles were also divided into three groups according to the proportional FSH used per harvested oocyte (1–84 *IU*, 85–237 *IU* and ≥238 *IU*).

### Statistics:

The statistical significance of the differences between the frequency distributions of MN embryos and embryo cohorts with MN embryos was tested with the Pearson’s chi-square test. At first, univariable binary logistic regression was used to assess the associations between MN embryos and independent variables (Female and male ages, number of oocytes and total and proportional FSH consumption). Multivariable binary logistic regression included the independent variables showing significance in the univariate analysis. Odds ratios (ORs) and 95% confidence intervals (95% CIs) were calculated to quantify the significant associations. The p-values less than 0.05 were interpreted as significant. All statistical analyses were carried out using SAS version 9.4 (SAS Institute, Cary, NC, USA).

### Ethical approval:

The study had approval from the Joint Ethics Committee of Turku University Central Hospital, Turku, Finland (117/1801/2013).

## Results

### Occurrence of MN embryos before freezing (D2), after thawing (D2t) and overnight culture (D3t):

Multinucleation was detected in at least one embryo in 259 out of 415 (62.4%) embryo cohorts on D2. On D2t, MN embryos were detected in 52 out of 320 embryo cohorts (16.3%). On D3t, multinucleation was observed in 84 of the 265 cohorts (31.7%), showing a lower occurrence than on D2 (p<0.001). Presence of MN embryos in a D2 embryo cohort was not associated with de novo MN embryo formation on D2t (p=0.113) or on D3t (p= 0.845) (Figure 1). Moreover, 572 embryos out of all 3430 embryos were MN (16.7%) on D2. Of the 1060 survived embryos, 61 were MN (5.8%) on D2t. On D3t, 108 out of 771 (14.0%) embryos were MN (Figure 1). Multinucleation occurred with similar frequency in cycles stimulated with antagonist or agonist protocol (p=0.800).

### Multinucleation in embryo cohorts, female age and ovarian response:

The mean age of the women at the time of ovum pick up was 33±4 years (Range 23–40). On D2, increasing female age was associated with a lower frequency of MN embryos per an IVF/ICSI cycle; the increase of four years gave an OR of 0.69 (95% CI 0.56–0.85, p<0.001) for MN embryos. Further, there was an interaction between the presence of MN embryos in an embryo cohort and female age, and the number of harvested oocytes (p<0.001); the frequency of MN embryos was the lowest in older women with a low number of collected oocytes.

For a more detailed analysis, three female age categories (20–29 years, 30–35 years and 36–40 years) were divided into subgroups based on the number of retrieved oocytes (1–8; 9–17; ≥18) (Table 1). MN embryos were most frequently observed in the age group of 20–29 years and in this age group, the occurrence was not associated with the number of retrieved oocytes (Table 1). In the age categories of 30–35 years and 36–40 years, the occurrence of MN embryos on D2 was the lowest when the number of harvested oocytes was less than eight. In these age groups, an association with high ovarian response and blastomere multinucleation was seen. Female age or number of retrieved oocytes was not associated with the multinucleation in embryo cohorts after thawing on D2t (p=0.964 and p=0.551, respectively) or D3t (p=0.806 and p=0.189, respectively), and there was no interaction effect (D2t p=0.926 and D3t p=0.405) in any age group.

**Table 1.** Multinucleation on day-2 of embryo culture in relation to female age and number of obtained oocytes per ovum pick up

**Female age (years)**	**No. of IVF/ICSI cycles**	**No of oocytes 1–8**	**No of oocytes 9–17**	**No of oocytes >17**	p ^**^

		% (n/n) ^*^	% (n/n) ^*^	% (n/n) ^*^	

		**n=95**	**n=214**	**n=106**	
**20–29**	87	69.2 (9.13)	72.5 (29.40)	79.4 (27.34)	0.702
**30–35**	207	38.5 (15.39)	69.2 (81.117)	78.4 (40.51)	<0.001
**36–40**	121	20.9 (9.43)	59.7 (34.57)	71.4 (15.21)	<0.001

* No. of embryo cohorts with multinucleation/all cohorts in a subgroup in parentheses.

** Chi Square test. IVF=*In vitro* fertilization, ICSI= Intracytoplasmic Sperm Injection

### Multinucleation in embryo cohorts, female age and FSH consumption:

On D2, the probability of multinucleation decreased with an increase in the total and proportional FSH consumption (Table 2). In contrast, no such correlation was seen on D2t or D3t (Table 2). However, when the study group was further divided into age categories (Table 3), an interaction between female age, proportional FSH consumption, and presence of MN embryos was seen on D2 (p=0.003); the occurrence of MN embryos was independent of the proportional FSH consumption in the age group of 20–29 years, but in women aged 30–35 years and 36–40 years, MN embryos were more common when the proportional dose of FSH was low. A similar association was detected in the age group of 36–40 years both on D2t and D3t (Table 3).

**Table 2.** Multinucleation in relation to total and proportional FSH consumption in IVF/ICSI cycles on day-2 of embryo culture (D2), after thawing (D2t) and on day-3 of embryo culture after thawing (D3t)

**Time point of embryo evaluation**		**Embryo cohorts without multinucleation**	**Embryo cohorts with multinucleation**	p ^*^	**OR (95% CI)**
**D2**					
	No. of IVF/ICSI cycles	146	244		
	FSH dose (IU)	2167.4±899.1	1815.5±721.4	<0.001	0.65 (0.52–0.80)
	Prop. FSH ^**^	275.4±237.2	144.6±116.9	<0.001	0.36 (0.36–0.50)
**D2t**					
	No. of IVF/ICSI cycles	255	46		
	FSH dose (IU)	1980.7±815.5	1882.3±761.0	0.447	0.89 (0.63–1.23)
	Prop. FSH ^**^	199.5±190.5	167.3±193.9	0.296	0.81 (0.55–1.20)
**D3t**					
	No. of IVF/ICSI cycles	177	81		
	FSH dose (IU)	1987.7±815.5	1893.2±763.0	0.379	0.88 (0.67–1.17)
	Prop. FSH ^**^	201.9±179.7	158.3±144.4	0.062	0.71 (0.49–1.02)

Numbers are mean±SD;

* Logistic regression analysis;

** Proportional FSH (IU/oocyte); IVF/ICSI cycles stimulated with corifollitropin excluded from the analysis (n=25); FSH= follicle stimulating hormone, IVF= *In vitro* fertilization, ICSI, intracytoplasmic sperm injection

**Table 3.** Multinucleation in relation to proportional FSH consumption and female age in IVF/ICSI cycles on day-2 of embryo culture (D2), after thawing (D2t), and on day-3 of embryo culture after thawing (D3t)

**Time point of embryo evaluation**	**Female age (years)**	**No. of IVF/ICSI cycles**	**Categories of proportional FSH consumption, % (number of embryo cohorts with multinucleation/all cohorts in the subgroup)**			p ^*^	**OR (95% CI)**

			**1–84 IU/oocyte**	**85–237 IU/oocyte**	**≥238 IU/oocyte**		
**D2**							
	20–29	86	81.1 (30.37)	71.1 (27.38)	72.7 (8.11)	0.856	0.95 (0.53–1.69)
	30–35	195	88.2 (45.51)	63.6 (68.107)	40.5 (15.37)	<0.001	0.25 (0.13–0.47)
	36–40	109	75.0 (6.8)	59.6 (31.52)	28.6 (14.49)	<0.001	0.35 (0.20–0.63)
**D2t**							
	20–29	73	25.8 (8.31)	9.4 (3.32)	20.0 (2.10)	0.334	1.33 (0.75–2.35)
	30–35	142	12.8 (5.39)	13.2 (10.76)	11.1 (3.27)	0.682	0.87 (0.44–1.71)
	36–40	86	37.5 (3.8)	23.1 (9.39)	7.7 (3.39)	0.033	0.33 (0.12–0.91)
**D3t**							
	20–29	60	46.4 (13.28)	16.0 (4.25)	57.1 (4.7)	0.836	0.89 (0.30–2.67)
	30–35	120	28.1 (9.32)	29.2 (19.65)	21.7 (5.23)	0.503	0.83 (0.49–1.42)
	36–40	78	75.0 (6.8)	34.3 (12.35)	25.7 (9.35)	0.028	0.47 (0.20–0.90)

* Logistic regression analysis; IVF/ICSI cycles stimulated with corifollitropin excluded from analysis (n=25); IVF=*In vitro* fertilization, ICSI=Intracytoplasmic Sperm Injection, FSH=Follicle Stimulating Hormone

## Discussion

To our knowledge, this is the first study that systematically assessed the de novo occurrence of MN embryos after embryo cryopreservation with slow freezing and the interaction between multinucleation, female age and ovarian response to gonadotropins. MN embryos were frequently seen in good-quality D2 embryo cohorts suitable for freezing, but the occurrence was less prevalent among frozen-thawed D3 embryos. The presence of MN embryos prior to freezing was not associated with increased de novo MN formation in sibling embryos during overnight post-thaw culture. Interestingly, the multinucleation rate was the highest in fresh D2 embryo cohorts of young women, regardless of ovarian response during COS, and in older women with a good ovarian response to COS. Intriguingly, the subjects in our study represented good prognosis patients in assisted reproductive treatment (ART). Further, the presence of multinucleation in embryo cohorts was similar irrespective of the ovarian stimulation protocol.

Little is known of the multinucleation rate in frozen-thawed embryos (14, 17). While the cooling of oocytes is reported to induce spindle disorganization (12, 13), the question of whether embryo freezing is a risk factor for post-thaw MN embryo formation still remains to be answered. The impact of freezing on the developmental potential of the embryos is most reliably seen after extended embryo culture (18–20). Consequently, if freezing would disturb nucleus reconstruction, it should become evident after the resumption of mitosis. In our study, the occurrence of de novo multinucleation after post-thaw culture was significantly lower than before freezing, and it corresponded with the de novo multinucleation rate previously reported in fresh D3 embryo cohorts (6, 21). Thus, slow freezing appears safe in terms of embryo multinucleation. Nevertheless, prospective studies comparing multinucleation in fresh and frozen-thawed D3 embryos are needed to further evaluate the relationship between slow freezing and MN embryo formation.

Further, it was found that post-thaw multinucleation occurred irrespective of the presence of MN embryos before freezing. Fresh and frozen-thawed MN embryos have impaired pregnancy potential (19, 22–24), but data on the developmental capacity of the sibling embryos in fresh IVF/ICSI cycles are conflicting (7, 8, 17). There are no previous reports in the literature regarding whether the presence of MN embryos before freezing is indicative of the overall developmental potential of the embryo cohort after freezing and thawing. Our results imply that the presence of MN embryos before cryopreservation does not increase the risk of multinucleation in sibling embryos after thawing, and therefore, if blastocyst culture is not possible, cleavage-stage sibling embryos can be safely cryopreserved.

MN embryos were frequently seen in IVF/ICSI cycles among women less than 30 years, regardless of the number of harvested oocytes, whereas multinucleation was associated with a higher response to ovarian stimulation in older age groups. In women over 35 years, the association between FSH sensitivity and multinucleation was also sustained over the freezing-thawing process. In the literature, female age, as a predisposing factor to blastomere multinucleation, has yielded contradictory results. In general, no association has been found (6–8, 11), apart from two studies by Moriwaki et al. (2004) (9) and Balakier et al. (2016) (10) showing a higher occurrence of MN embryos in older women. However, in these studies, the impact of female age on multinucleation was evaluated independent of other factors affecting ovarian response to COS, whereas our study is the first to explore multinucleation and its interaction between female age and ovarian response to COS. These results add to the evidence that multinucleation is associated with ovarian sensitivity to hormonal stimulation (4, 5, 7). The relationship between MN embryos and female age and ovarian response further suggests that the inclination to multinucleation is derived from conditions that occur during oocyte maturation or patient characteristics.

Advanced female age is particularly known to have a strong association with an increasing number of chromosomal errors and deterioration in oocyte quality. Interestingly, in this study, multinucleation was found to be most prevalent in the youngest age category of women with embryos eligible for cryopreservation. First, studies using the fluorescence in situ hybridization found a strong association between chromosomal abnormalities and multinucleation, as up to 80% of the cleavage-stage MN embryos were reported to be aneuploid (3, 16). However, recent aneuploidy records gained from blastocyst stage laser-assisted trophectoderm biopsies have demonstrated that the aneuploidy rate at the blastocyst stage in normally developing MN embryos does not differ from that of mononucleated embryos (10, 25). This has contradicted the hypothesis that multinucleation is a sign of aneuploidy and, thus, a definite indicator of poor embryo quality. Instead, multinucleation has been suggested to be more likely a consequence than a cause of chromosomal abnormalities and acts as a safeguard against aneuploidy (26). This hypothesis is based on observations that early cleavage-stage embryos are, in general, vulnerable to defective spindle assembly and mosaicism due to immature cell cycle checkpoints (27). Further, blastocysts derived from MN embryos produce live births with a relatively high rate (10, 17, 19). Therefore, multinucleation has been suggested to reflect an active self-correction process for mitotic aneuploidy errors in cleavagestage embryo (23, 28).

Only embryo cohorts including embryos eligible for cryopreservation were included in our study, and therefore, the study design illustrates MN formation in good-quality embryo cohorts. Our results showed that within this good prognosis cohort of women, multinucleation was also common in high-responding women in advanced age groups. Sekhon et al. (2017) (29) demonstrated that the number of collected oocytes and the gonadotropin dose during COS in normal responders were not related to an increase in aneuploidy rate. This further highlights the hypothesis that multinucleation is a repair process in the good prognosis patient group with normal ovarian reserves and overall good-quality embryo cohort. All in all, our findings more likely support the prevailing theory that multinucleation represents active repair mechanisms of abnormal chromosomal contents of blastomeres to restore embryo viability rather than poor quality of embryo.

The strength of our study was in embryo screening, which was performed systematically through the cooperation of two observers. Our results are in line with previous studies on fresh IVF/ICSI cycles reporting multinucleation of 6%–26% in day two embryos with sequential screening (4–7) and a frequency of 15%–25% in four-cell stage embryos during continuous embryo monitoring (10, 23, 24, 30). However, conventional sequential embryo screening may underestimate the rate of transient nuclear abnormalities. Even though there was no time-lapse monitoring, the conventional screening of embryos was based on a controlled double evaluation and joint consensus, minimizing interobserver variability. Although timelapse monitoring is an excellent method for studying embryo morphokinetics, its use is currently limited by high costs. Our study thus provides data that reflect clinical practice.

Today, vitrification has mainly replaced the traditional slow-freezing protocol because of the simplicity of the procedure and better clinical outcomes (31). It should be noted that the results from studies with slow freezing are not directly applicable to vitrification. Therefore, future studies evaluating the effect of vitrification on MN are warranted.

## Conclusion

In summary, the presence of MN embryos prior to freezing was not associated with blastomere multinucleation in sibling embryos after thawing. All in all, multinucleation in good-quality embryo cohorts was less common after thawing than before embryo cryopreservation. Our findings add to the evidence that the risk for MN embryos is associated with factors related to high ovarian response to hormonal stimulation – such as young age, FSH sensitivity and the higher number of obtained oocytes among women with advanced age. This supports the hypothesis that embryo multinucleation may represent an active repair mechanism of chromosomal errors in embryos rather than distinctly poor quality of embryo. Embryo freezing is increasing in popularity among ART treatments and the debate on the “freeze-all” policy is gaining interest. At the same time, however, concerns for the possible negative effects of cryopreservation on embryos have been expressed. The low frequency rate of MN embryos after thawing suggests the safety of slow freezing also in terms of nuclear reconstruction, but studies comparing multinucleation in fresh and frozenthawed D3 embryos are needed.
